# Clinical Outcomes of Machine Perfusion and Temperature Control Systems in Heart Transplantation: Where We Stand

**DOI:** 10.3390/jcm14041152

**Published:** 2025-02-11

**Authors:** Umar Nasim, Ander Dorken-Gallastegi, Peter Dadson, Yeahwa Hong

**Affiliations:** 1 Department of Biomedical Engineering, Carnegie Mellon University, Pittsburgh, PA 15213, USA; unasim@andrew.cmu.edu (U.N.); dorkengallastegia@upmc.edu (A.D.-G.); pdadson@andrew.cmu.edu (P.D.); 2 Department of Cardiothoracic Surgery, University of Pittsburgh Medical Center, Pittsburgh, PA 15213, USA; 3 Department of Surgery, University of Pittsburgh Medical Center, Pittsburgh, PA 15213, USA

**Keywords:** heart failure, heart transplantation, heart preservation, ex vivo machine perfusion, post-transplant outcomes

## Abstract

Heart transplantation remains the preferred treatment for carefully selected patients with end-stage heart failure refractory to medical therapy. Advances in donor management, organ preservation, donor and recipient selection, immunosuppressive strategies, and mechanical circulatory support have significantly improved the safety and efficacy of heart transplantation. However, the persistent shortage of donor hearts and their limited preservation period continues to restrict access to this lifesaving procedure. The advent of innovative machine perfusion and temperature control systems for heart allograft preservation offers a promising avenue to address these challenges. These technologies aim to extend preservation times and enable the use of extended-criteria donors, thereby expanding the donor pool. In this review, we examine the outcomes from clinical trials, registry data, and single-center studies, utilizing the TransMedics Organ Care System Heart, Paragonix SherpaPak Cardiac Transport System, and XVIVO Heart Preservation System. As the field of heart transplantation evolves to accommodate longer ischemia times, expand organ sharing, and utilize donors previously considered marginal, the integration of these advanced technologies will be essential for optimizing post-transplant outcomes.

## 1. Introduction

Advanced heart failure is a condition characterized by impaired ventricular function and chronic systemic malperfusion [1,2]. Many patients with this condition experience progressive deterioration despite receiving guideline-directed medical therapy. For these individuals, advanced therapeutic options are limited to heart transplantation or mechanical circulatory support (MCS), such as durable left ventricular assist devices (LVADs). Heart transplantation remains the gold-standard treatment for advanced heart failure, offering superior outcomes in both survival and quality of life [3]. Although many patients are in need of a heart transplantation, only 4500 new patients are added to the waiting list each year in the United States due to the persistent shortage of donor hearts [4]. Furthermore, the number of heart transplants performed annually in the United States has increased recently, but it remains limited to approximately 4000 procedures [4].

Static cold storage (SCS) has been the standard method for heart preservation prior to transplantation since the inception of this procedure. This approach involves storing the heart in a cardioprotective solution on ice but is significantly limited by the safe storage duration and diagnostic utility. The detrimental effects of SCS beyond four hours are well-documented, restricting the distance a donor heart can travel to reach a matching recipient [5]. Additionally, SCS prevents the transplant team from assessing the viability of the graft prior to transplantation. A cold, non-perfused heart does not provide any useful information regarding its ability to function after reperfusion in the recipient.

Advanced perfusion and temperature control systems have been developed to overcome the limitations of traditional heart preservation methods in transplantation [6]. Technologies such as the TransMedics Organ Care System (OCS) Heart, the XVIVO Heart Preservation System (XHPS), and the Paragonix SherpaPak Cardiac Transport System (SCTS) have been approved for clinical use and are increasingly adopted in practice [6]. In this review, we examine the current clinical literature evaluating the impact of these systems on heart transplantation, highlighting their strengths, limitations, and future directions for advanced heart preservation.

## 2. Methods

### 2.1. Literature Search

**Figure 1:**
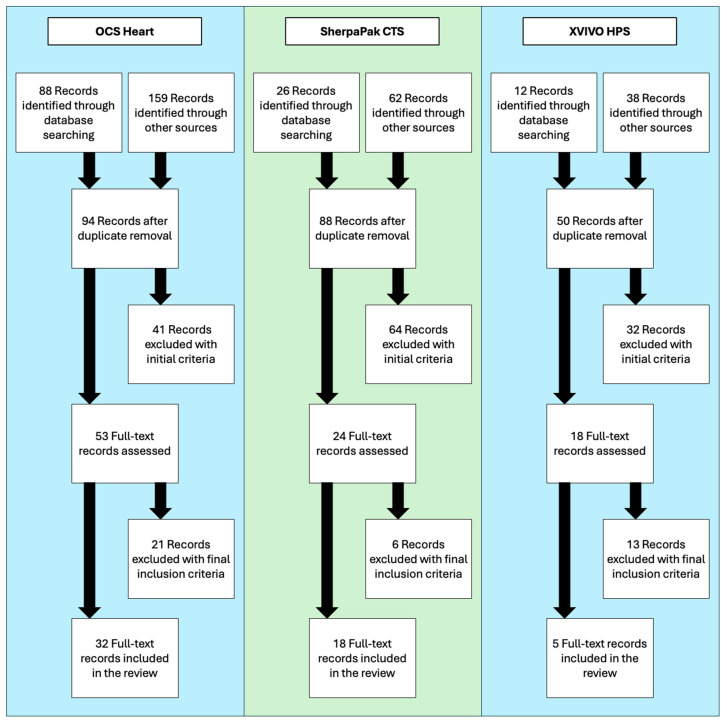
Flowchart illustrating the literature review and selection process for each heart preservation method. Abbreviations: Organ care system (OCS), Cardiac Transport System (CTS), Heart Preservation System (HPS).

PubMed, Scopus, and Google Scholar were queried with the terms “heart transplantation” and either “Organ Care System”, “SherpaPak” or “XVIVO” to generate the initial list of records. The reference lists from the included studies were reviewed to identify potentially relevant records. The titles and abstracts of these studies were used to remove articles based on the initial exclusion criteria. A full-text review of the remaining records was performed to select the final list of the included studies. These exclusion and inclusion criteria are detailed in Section 2.2, and the summary of the study inclusion flowchart is shown in Figure 1.

### 2.2. Selection Criteria

The initial exclusion criteria were applied to remove irrelevant records: (1) studies not published in or translated to English; (2) preclinical studies; and (3) book chapters, editorials, and commentaries. The final inclusion criteria applied during the full-text review required that the studies (1) were published in a peer-reviewed journal, (2) evaluated clinical outcomes after heart transplantation, and (3) included one of the three novel preservation systems as a study group.

## 3. TransMedics Organ Care System Heart

The Organ Care System (TransMedics, Andover, MA, USA) is the only commercially available normothermic ex vivo machine perfusion platform for resuscitation, assessment, and preservation of donor hearts. This system received the European conformity (CE) mark for clinical use in Europe in 2006, and the first clinical use was described at the Heart Center North Rhine-Westfalia, Germany, in the same year [7]. Approval for clinical use in the United States by the Food and Drug Administration (FDA) was not received until 2021 [8].

**Figure 2:**
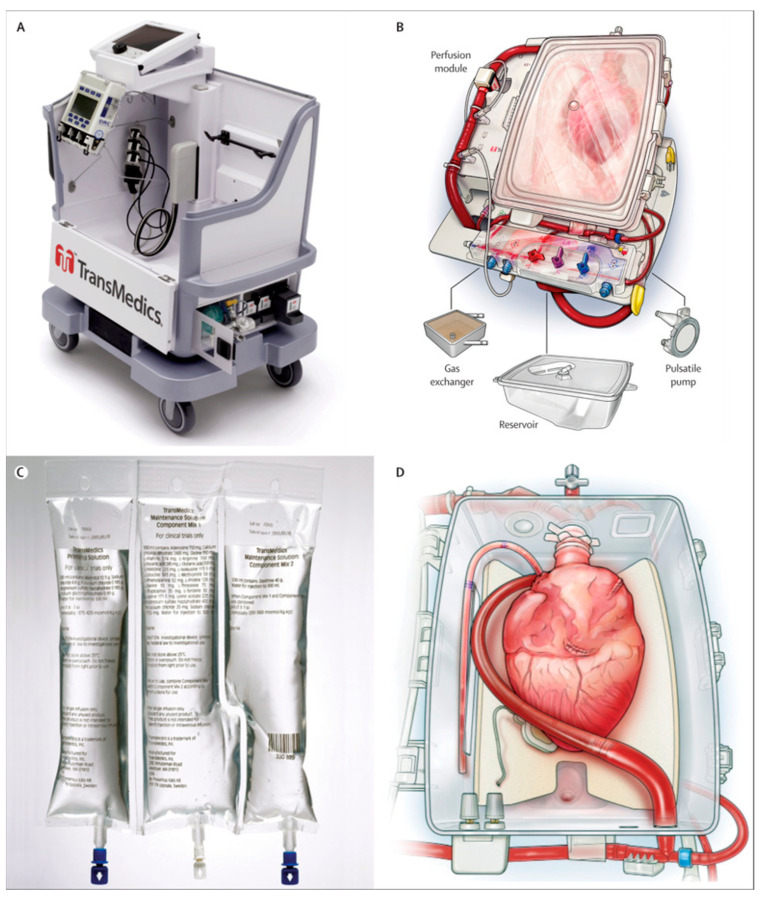
Overview of the TransMedics Organ Care System (OCS) Heart with the (**A**) integrated heart perfusion system cart, (**B**) heart chamber with circuit components, manifold access, and monitoring system, (**C**) OCS priming and perfusion solution, and (**D**) heart positioning with aortic, pulmonary artery, and left ventricular attachments. Figure from Ardehali, et al. 2015 [9].

The OCS features a pulsatile blood pump, oxygenator, blood reservoir, perfusion module, and heart preservation compartment (Figure 2) [9]. Following cardioplegia administration and subsequent procurement, the right atrium is closed, and an aortic cannula, pulmonary artery cannula, and left ventricular venting cannula are placed in the donor heart. The system delivers an oxygenated blood-based perfusate to the aortic root through the aortic cannula for antegrade pressure-controlled coronary perfusion. The right ventricle ejects venous blood from the coronary sinus through the pulmonary artery cannula into the blood reservoir, and the left ventricle remains unloaded. The perfusate is composed of 1.2–1.5 L of donor blood collected before procurement and mixed with the proprietary OCS heart solution containing electrolytes, osmotic agents, and nutrients to support metabolism [9].

The OCS allows for ex vivo functional assessment of donor hearts to facilitate a final determination regarding the usability of the graft. The venous and arterial lactate levels and coronary perfusion flow rate and pressure are used to quantitatively assess the graft. A visual examination of ventricular contractility is also performed, and the decision to utilize the organ is ultimately at the discretion of transplant physicians. Upon arrival at the recipient’s center and acceptance for use, the heart is once again arrested with cardioplegia and disconnected from the OCS for transplantation [10].

### 3.1. Clinical Trials Evaluating OCS Heart

Multiple clinical trials have evaluated the OCS for heart allograft preservation and evaluation prior to transplantation. Over the past 10 years, these trials evaluated whether (1) normothermic heart perfusion was safe and effective with standard donor criteria, (2) the OCS was a useful tool to evaluate extended criteria donors (ECDs) prior to transplantation, and (3) heart allografts were viable following donation after circulatory death, enabling the option of donation after circulatory death (DCD) heart transplantation. In this section, we will evaluate the design, outcomes, and impact of these studies on clinical practice. We will also discuss the limitations and future directions of the OCS Heart.

#### 3.1.1. PROCEED II Trial

The Ex vivo Perfusion of Donor Hearts for Human Heart Transplantation (PROCEED II) trial was a prospective, open-label, multicenter, randomized clinical trial to assess the non-inferiority of the OCS compared to SCS, as summarized in Table 1 [9]. Only brain-dead donors were included, and strict inclusion and exclusion criteria were applied based on standard donor, recipient, and expected transplant risk factors. The primary outcome was 30-day patient and graft survival, and secondary outcomes included cardiac-related serious adverse events, severe rejection, and intensive care unit (ICU) length of stay. The trial enrolled 130 patients (OCS n = 67, SCS n = 63) between 2010 and 2013 across 10 United States and European heart transplant centers. The primary analysis demonstrated that the OCS was non-inferior to SCS (94% vs. 97%, *p* = 0.36) in the as-treated comparison. Furthermore, the rates of secondary outcomes were similar between the two groups (all, *p* > 0.05) [9]. The OCS group possessed significantly longer preservation times (324 vs. 195 min, *p* < 0.001) and shorter cold ischemic times (113 vs. 195 min, *p* < 0.001). Five hearts assigned to the OCS group were discarded due to rising lactate levels in the perfusate during ex vivo perfusion (n = 4) and friability of the aortic tissue, precluding cannulation to the OCS platform (n = 1) [9].

Two additional retrospective studies were conducted using patients enrolled in the PROCEED-II trial at a single institution to evaluate long-term post-transplant survival (n = 19 for both OCS and SCS groups). No significant differences were observed in the 2-year (OCS 72.2% vs. SCS 81.6%, *p* = 0.38) and 8-year (OCS 57.9% vs. SCS 73.7%, *p* = 0.24) post-transplant survival rates between these two smaller cohort studies. The rates of cardiac allograft vasculopathy (CAV) and non-fatal major adverse cardiac events (NF-MACE) were also comparable between the OCS and SCS groups [34,35].

Further analysis of patients from the PROCEED-II trial was conducted to assess the development of CAV using intravascular ultrasound (IVUS). The study evaluated and compared the progression of coronary intimal thickening within the first year after transplantation between the OCS (n = 5) and SCS (n = 13) groups. No significant differences were observed in post-transplant survival or CAV progression between the two groups [36].

The PROCEED-II trial played a pivotal role in demonstrating that isolated normothermic perfusion of a heart allograft is a safe and effective preservation method prior to transplantation with standard selection criteria (e.g., non-marginal donors). The utility of perfusate lactate levels for determining the viability of the allograft during the perfusion period was well supported by this study. Each of the donor hearts discarded for hyperlactatemia was found to have notable pathological findings, including myocardial contusion, myocardial necrosis and scarring, and aortic regurgitation that prevented stable perfusion on the OCS [9]. Later studies built on this work by leveraging the OCS to assess donor heart function immediately before implantation, particularly in the context of ECD hearts and, ultimately, DCD heart transplantation.

#### 3.1.2. EXPAND and EXPAND-CAP Trial

The OCS Heart Expanded Criteria Donor Hearts for Transplantation (EXPAND) trial investigated the use of the OCS platform for the ex vivo resuscitation, assessment, and preservation of ECD hearts (marginal donors) from donation after brain death (DBD) donors, summarized in Table 1. This prospective, single-arm, multicenter clinical trial enrolled 173 recipients of marginal donor hearts across transplant centers in the United States from 2015 to 2021. Marginal donor criteria were established by an independent expert panel, defining them based on factors such as donor cross-clamp time ≥ 4 h or ≥2 h with additional risk factors, including donor age, drug use, coronary disease, and suboptimal ventricular structure and function [37]. No direct control group was used in this trial, as preserving marginal donor hearts with SCS was considered unethical due to anticipated poor outcomes. Instead, contemporary standard criteria heart transplantation with SCS served as a reference group. This study aimed to define criteria for utilizing a donor heart after normothermic perfusion on the OCS platform; these are arterial lactate < 5 mmol/L and stable coronary perfusion pressure and flow. However, the final decision on heart utilization remained at the discretion of the transplant surgeon [37].

The EXPAND trial included an additional cohort from the EXPAND-Continued Access Protocol (CAP), bringing the total to 173 donor hearts placed on the OCS [11]. The results of the EXPAND trial with the additional patients from the EXPAND-CAP will be collectively analyzed and referred to as the EXPAND-CAP trial for the remainder of this review. Of these, 86.7% were utilized for transplantation (n = 150). The average total cross-clamp time of the transplanted hearts exceeded 6 h (374.3 ± 88.6 min), and the OCS perfusion time was typically greater than 4 h (271.9 ± 80.4 min). The primary reason for the exclusion of 23 (13%) hearts after ex vivo perfusion with OCS was elevated lactate levels. These hearts typically exceeded the 5 mmol/L criteria for discard, compared to the average endpoint lactate level in the transplanted hearts of 3.08 ± 1.13 mmol/L [11].

The recipients of ECD hearts preserved with the OCS surpassed the pre-determined effectiveness goal, defined as 65% of patients achieving 30-day survival and freedom from severe primary graft dysfunction (PGD) as defined by the International Society of Heart and Lung Transplantation (ISHLT) criteria (92%, *p* < 0.0001). The rate of serious graft-related adverse events was 17%, and moderate-severe PGD occurred in 16% of patients. Overall post-transplant outcomes were excellent, demonstrating survival rates of 89.3% after 1 year and 86.0% after 2 years [11].

The EXPAND-CAP trial represented a significant milestone in heart transplantation, showcasing the effective assessment and transportation of marginal donor hearts using normothermic ex vivo perfusion with the OCS platform. This advancement has enabled the inclusion of extended selection criteria hearts into the donor pool, addressing the critical mismatch between the growing number of patients on the heart transplant waitlist and the limited availability of donor hearts. Results from EXPAND-CAP indicate that marginal donor hearts, previously considered unsuitable for transplant using SCS standards, can be safely utilized following perfusion and ex vivo selection with OCS. A recent analysis of the United Network for Organ Sharing (UNOS) registry in 2021 revealed that applying extended donor criteria similar to those in the EXPAND trial could expand the donor pool by 8637 patients between 2015 and 2019, highlighting the significant potential for increasing the donor heart pool [37]. As the heart transplant community continues to interpret and discuss the implications of the EXPAND trial, the full clinical impact of the widespread application and operationalization of the OCS platform is not yet fully understood.

#### 3.1.3. DCD Heart Trial

The Clinical Trial to Evaluate the Safety and Effectiveness of The Portable Organ Care System Heart For Resuscitation, Preservation and Assessment of Hearts From Donors After Circulatory Death (DCD Heart Trial) was a non-inferiority trial designed to evaluate outcomes after DCD heart transplantation based on promising single-center experiences in Australia and the United Kingdom. This prospective, multicenter, randomized, unblinded controlled trial compared standard risk criteria DBD heart donors preserved with SCS (DBD-SCS) to DCD donors perfused with the OCS (DCD-OCS). The inclusion criteria for the DCD-OCS donors were 18–49 years old and a functional warm ischemic time (FWIT) of less than 30 min [12,38]. FWIT was defined as the time from decreased perfusion parameters (systolic blood pressure < 50 mmHg or arterial saturation < 70%) to donor cross-clamp and the administration of cold cardioplegia. The primary endpoint of the study was the risk-adjusted 6-month survival rate. Secondary endpoints included donor heart utilization on the OCS, 1-year post-transplant survival, and freedom from serious graft-related adverse events [12].

Each group was initially allocated 90 patients, but after exclusions due to protocol violations, the final as-treated population included 80 recipients of DCD-OCS hearts and 86 recipients of DBD-SCS hearts. A total of 11 donor hearts placed on the OCS were discarded (89% utilization). These allografts were declined for transplantation due to increasing lactate levels despite appropriate perfusion parameters (n = 5), rising lactate levels with poor contractility evaluation (n = 5), or poor contractility evaluation alone (n = 1). The mean cold ischemic and normothermic perfusion times were not reported [12].

The unadjusted (DCD-OCS 95% vs. DBD-SCS 89%, *p* < 0.001 for noninferiority) and risk-adjusted (DCD-OCS 94% vs. DBD-SCS 90%, *p* < 0.001 for noninferiority) 6-month survival demonstrated that DCD heart transplantation with evaluation on the OCS was safe. Notably, the rate of moderate or severe PGD was substantially higher in the DCD-OCS cohort than the DBD-SCS (22% vs. 10%, no *p*-value reported). The 1-year survival rate of the DCD-OCS group was nominally higher than that of the DBD-SCS group (93% vs. 85%) despite the increased rate of PGD [12],

This randomized multicenter trial demonstrated that DCD heart transplantation was non-inferior to standard-of-care heart transplantation when using the OCS platform for the preservation and evaluation of the allograft. This finding supports the early single-center DCD heart transplantation experiences from Australia and the United Kingdom, which will be discussed in Section 3.2.2. A new analysis will assess the long-term post-transplant survival and complications in this patient population with 5 years of follow-up.

#### 3.1.4. Limitations of the OCS Heart Clinical Trials

The PROCEED-II, EXPAND-CAP, and DCD Heart clinical trials highlighted the safety and efficacy of the OCS Heart system. These studies exemplified the hallmarks of rigorous clinical research, including prospective design, multicenter inclusion, and pre-defined statistical methods. However, several significant limitations remain, stemming from the trials’ implementation and real-world constraints. First, randomization was not feasible in the EXPAND-CAP and DCD Heart Trials due to ethical considerations [11,12]. However, the outcomes of standard criteria donors preserved with SCS provided a useful benchmark. Second, variability in center practices and individual physician preferences may influence outcomes. In the PROCEED-II trial, the center-preferred cardioplegic solution was used during heart procurement and to arrest the heart allografts removal from the OCS [9]. Additionally, manual control of coronary flow rate and adenosine infusion were performed to maintain appropriate perfusion conditions. In contrast, the EXPAND-CAP and DCD Heart trials standardized this process by adopting the del Nido solution and automated control of coronary perfusion by the OCS platform [11,12]. Despite this standardization, other differences, including post-transplant management protocols, resource availability, and population demographics, may have unknown effects on outcomes.

Third, variability in the criteria for allograft discard or utilization within the OCS platform remains a key limitation. Across these trials, the perfusate lactate levels were the primary metric guiding this decision. However, a threshold of lactate of <5 mmol/L was used for accepting DBD donors in the PROCEED-II and EXPAND-CAP trials but “stable or down-trending” lactate levels were used for the DCD donors in the DCD Heart Trial [9,11,12]. This raises the question of whether the absolute lactate value, which can be artificially elevated or depressed by the baseline conditions in the perfusate, or the behavior of the lactate profile during perfusion is a superior predictor of allograft viability. Despite these criteria, approximately half of allografts discarded in these studies were attributed to clinical judgment by the transplant team. This failsafe is critical to address unforeseen issues with the donor heart and minimize potential harm. However, the absence of a quantitative risk score or standardized guidelines for graft evaluation on the OCS highlights a critical gap in current practice.

### 3.2. Single-Center Experience with the OCS Heart

#### 3.2.1. High-Risk Donor and Recipient Profiles

Several centers worldwide have reported their experiences and outcomes using the OCS Heart platform for DBD heart transplantation across various donor and recipient risk profiles. The first report using the OCS Heart to preserve and evaluate ECD for heart transplantation was published before the PROCEED II trial. This single-center experience by García Sáez et al.in 2014 evaluated 30 recipients of DBD-OCS heart allografts in the United Kingdom [13]. The donors were classified as high-risk based on an expected preservation time exceeding 4 h, left ventricular ejection fraction less than 50%, mild left ventricular hypertrophy (>1.3 cm), or palpable coronary abnormalities without angiography. The recipient risk factors considered in this study were either pulmonary hypertension or bridge to transplantation (BTT) with a durable LVAD. No allografts were discarded from the OCS Heart, and the mean OCS Heart perfusion time was 285 ± 92 min. The 30-day survival rate was 100%, and moderate right ventricular graft dysfunction was observed (n = 5, 19.2%) [13]. This small study displayed the promise of the OCS Heart platform for ECDs and high-risk recipients, advocating for the greater utilization of this device to expand the donor pool.

A similar study by Sponga et al., performed in Italy and published in 2020, examined 5-year post-transplant outcomes in patients with ECD. The definitions of ECDs in this analysis were similar to those defined by García Sáez et al., but high-risk recipients were excluded. The donor hearts were preserved with either the OCS (n = 21) or SCS (n = 79) [14]. The OCS perfusion group achieved an 81% transplantation rate, with hyperlactatemia being the primary reason for discarding grafts. The overall rate of postoperative complications was lower in the OCS cohort (OCS 67% vs. SCS 87%, *p* = 0.04), defined as PGD requiring extracorporeal membrane oxygenation (ECMO), intra-aortic balloon pump (IABP), atrial fibrillation, renal or respiratory insufficiency, and acute rejection. The 5-year survival rate was also higher for the OCS patients (OCS 100% vs. 73%, *p* = 0.04). This study demonstrated that OCS provides superior outcomes to SCS for this vulnerable population [13]. Furthermore, a subsequent retrospective analysis by the same group in 2023 examined the outcomes of ECD hearts preserved with OCS that were transplanted into high-risk recipients. These high-risk recipients were older with higher rates of chronic renal dysfunction, liver dysfunction, and prior cardiac surgery. Despite these high-risk features, 1-year and 3-year survival rates were 83% and 72%, respectively [15].

A retrospective multicenter report from Germany also compared the use of OCS and SCS for ECDs with high-risk recipients, primarily redo-transplantation [16]. Thirty-day (OCS 92.4% vs. SCS 90.2%, *p* = 0.745) and one-year (OCS 89% vs. SCS 85%, *p* = 0.225) survival rates were comparable between the OCS and SCS groups. While post-transplant mechanical ventilation and the need for ECMO were similar, the OCS group exhibited a significantly lower incidence of post-transplant renal failure requiring dialysis (4.4% vs. 27.5%, *p* < 0.001).

Two studies further examined the impact of recipient MCS as a BTT with the OCS for donor heart preservation. Sponga et al. and Kaliyev et al. retrospectively analyzed patients at their respective centers who were bridged to heart transplantation with durable MCS. Sponga et al. (OCS n = 25, SCS n = 10) observed a significantly lower rate of PGD with the OCS compared to SCS (OCS 7% vs. SCS 42%, *p* = 0.03) [17]. Kaliyev et al. confirmed these findings, with a lower proportion of post-transplant ECMO support observed in the OCS group compared to the SCS group (OCS 24% vs. SCS 60%, *p* = 0.02) [18]. These results highlight the promising potential of OCS for preserving DBD hearts in patients bridged to transplantation with MCS devices, particularly in cases where prolonged ischemic times are anticipated due to extended recipient procedures, such as durable LVAD explantation during the cardiectomy.

Prolonged ischemic time with OCS use is another important risk factor associated with the OCS platform evaluated by multiple studies. During normothermic perfusion, the donor heart actively produces waste products and consumes the metabolites in the perfusate, which can lead to biochemical imbalances that may damage the donor organ if perfusion is extended. However, multiple case reports have demonstrated that in situations where prolonged perfusion is unavoidable and SCS is not an option, the OCS can safely preserve an allograft for up to 17 h with careful allograft management [19,20,21,24,25]. The independent impact of increasing preservation time with the OCS has not been formally investigated, as factors related to the donor heart and exact perfusion conditions may affect this result.

The use of OCS for ex vivo perfusion of pediatric cardiac allografts has also been explored, demonstrating that early post-transplant survival is comparable to that achieved with SCS [22]. This study utilized the standard OCS platform without any modifications for a pediatric donor, and a minimum recipient and donor weight of 15 and 45 kg, respectively, was applied. As a result, the weight distribution of the SCS cohort (n = 13) was 13 [IQR: 3.5–57] kg compared to the OCS cohort (n = 8) 36 [IQR: 17–89] kg (*p* = 0.48). Similarly, another center evaluated eight pediatric patients transplanted with hearts preserved with the OCS. Only donors > 40 kg were utilized, and the median recipient age was (13, range [9,10,11,12,13,34,35,36,37,38] years) and weight was (58.8, range [33.2–127.8] kg). Notably, 25% (n = 2) of transplantations were from DCD donors, and all patients survived to a median follow-up time of 11.9 months [26]. Additionally, OCS has been utilized for the transplantation of adult patients with end-stage congenital heart disease, with 11% post-transplant mortality within 30 days and 22% within 1 year of transplantation [23]. The primary reason for OCS utilization in these studies was the prolonged preservation time the system offered due to the expected extended duration of the recipient procedures.

#### 3.2.2. DCD Heart Transplantation

As demonstrated by the DCD Heart Trial, the OCS platform was a significant enabling technology for DCD heart transplantation. However, the promising post-transplant outcomes of this technique were first presented by single-center experiences from Australia and the United Kingdom. The study presented by Dhital et al. was the first case series of 3 DCD heart transplantations that utilized the OCS [27]. All donor warm ischemic times were <30 min, and OCS perfusion time was approximately 250 min. Following transplantation, 2/3 of the patients required temporary MCS, and all patients survived the first 30 days. A case series from the United Kingdom reported the experience of procuring and perfusing 8 DCD hearts with the OCS between 2017 and 2018 [28]. The median functional warm ischemia time was 28 min, and the median OCS perfusion time was 263 min. Of the eight hearts, seven were successfully transplanted. Post-transplant 30- and 90-day survival rates were 100% and 86%, respectively. There was a high incidence of extracorporeal life support requirement (3/7, 43%) [28].

One important consideration for this population is the value of lactate as a decision-making parameter. Multiple studies for DBD heart transplantation with the OCS utilize a threshold of serum lactate greater than 5 mmol/L as an absolute contraindication of donor heart utilization [11]. In the DCD Heart trial, the condition was changed to increasing lactate levels rather than a concentration cutoff [12]. However, a study performed by Cernic et al. investigated graft dysfunction and the need for post-transplant MCS based on OCS lactate levels for DCD hearts and found no predictive value associated with this variable [29]. This difference is important to consider and should be examined further to identify concrete acceptance and rejection of OCS heart allografts.

A retrospective single-center study from the United States compared cardiac MRI findings and clinical outcomes between different methods of allograft donation and preservation: DCD with OCS (n = 31); DBD with OCS (n = 16); and DBD with SCS (n = 38) [30]. The DCD OCS cohort had a significantly higher incidence of PGD (19.5%) compared to DBD OCS (0) and DCD SCS (5.3%). There was no difference in mortality or rejection among the groups, despite the increased rate of PGD. Cardiac MRI findings for ventricular wall sizes, vascular abnormalities, and pericardial effusion were not significantly different among the different groups.

#### 3.2.3. Advanced Use of the OCS Heart

The normothermic perfusion of the donor heart on the OCS approximates the native conditions of the organ. Therefore, various diagnostic and interventional procedures can be performed on the isolated heart without significantly altering existing in vivo techniques. Multiple examples of these interventions have been documented in case series and reports. One of the first single-center case reports of OCS highlighted a successful coronary angiography performed during heart perfusion, accomplished with standard techniques using the OCS access port [31]. A systematic review of these procedures revealed nine individual case reports, all demonstrating successful transplantation of six donor hearts with no allograft injury [32]. Additionally, other centers have showcased innovative methods of allograft analysis, such as IVUS cardiac magnetic resonance imaging and metabolomic profiling of cardiac energy reserves. While the clinical utility of these techniques remains a topic of debate, they represent valuable options uniquely enabled by the OCS platform. Further analysis of how new imaging modalities and metabolic markers correspond with post-transplant outcomes could inform a greater understanding of graft viability for transplantation beyond lactate levels.

The OCS platform has also enabled a novel method of heart transplantation developed by Stanford University, known as ‘beating heart’ transplantation with DCD donors. This method, first presented in 2023, utilizes a modified OCS to maintain uninterrupted coronary perfusion during recipient implantation [39,40]. In this approach, the donor heart is prepared for the transition to cardiopulmonary bypass perfusion in the operative field by placing a root cannula in the aorta, cardiotomy suction in the left and right atria, and epicardial pacing wires are sewn onto the heart’s surface. A cross-clamp is applied superior to the aortic vent, and cardiopulmonary bypass flow is initiated, allowing the heart to transition from the OCS without interruption. During the left atrial and aortic anastomoses, the heart is perfused and beats under the control of the epicardial pacemaker. After completing the aortic anastomosis, the cross-clamps are removed, and the root cannula serves as a root vent, continuing the procedure as normal. Outcomes following ‘beating heart’ transplantation have been promising, with an early case series of 10 patients who received DCD heart allografts. All 10 patients survived without PGD or subsequent graft failure, with a median follow-up of 97.5 days [IQR 14–293] [41]. A more recent analysis described outcomes for 24 patients receiving ‘beating heart’ transplantation from both DCD (87.5%) and DBD (12.5%) donors. With a median follow-up of 192 days [IQR 124–253.5], 95.8% survived, and no post-operative MCS was required [33].

### 3.3. Conclusions and Remaining Questions with the OCS Heart

The OCS is an essential tool for modern heart transplantation, expanding the potential donor pool and increasing access to this lifesaving therapy. These clinical studies have shown that the continued expansion of the characteristics of marginal donors does not adversely impact post-transplant outcomes as long as the performance of the organ on the OCS remains stable. It is important to monitor how the rates of graft utilization and discard evolve as the criteria for procurement and placement on the OCS change. However, many questions still remain regarding the optimal use and limitations of normothermic ex vivo heart perfusion. (1) What is the full clinical impact of the OCS platform in expanding the donor pool? (2) How can we establish concrete criteria for allograft utilization or discard based on donor and recipient risk factors? (3) What is the safe limit of normothermic perfusion with the OCS and how do donor characteristics impact this value? Finally, (4) what role can the OCS platform play in enabling advanced diagnostics or organ repair technologies during perfusion? Addressing these questions is essential for optimizing the potential of the OCS platform in heart transplantation.

## 4. SherpaPak Cardiac Transport System

**Figure 3:**
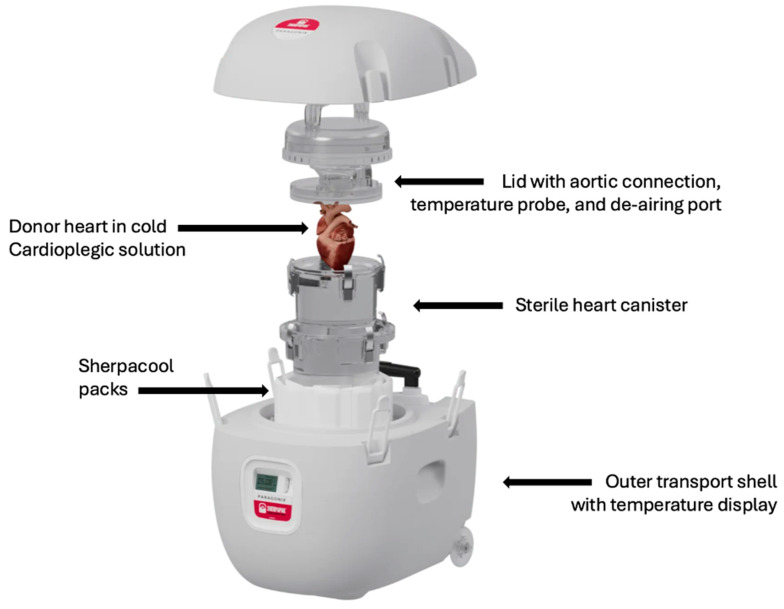
Cross-sectional view of the Paragonix SherpaPak Cardiac Transport System [43].

The SherpaPak Cardiac Transport System was developed by Paragonix Technologies Inc. (Braintree, MA, USA) and is FDA-approved and CE-marked for clinical use. This system utilizes disposable cooling packs instead of ice to maintain the donor heart and preservation solution between 4 °C and 8 °C (Figure 3). The first clinical use of this device was demonstrated by Naito et al. in 2020, where a donor heart traveled over 1100 miles with an ischemic time exceeding 5 h [42]. Since then, over 4000 donor hearts have been successfully preserved using the SCTS. The clinical efficacy of this system is extensively evaluated through the Global Utilization and Registry Database for Improved heArt preservatioN (GUARDIAN-Heart) clinical trial (NCT04141605) and multiple single-center analyses. Additionally, a recent randomized controlled trial (NCT05194514) conducted at the Cedars–Sinai Medical Center compared SCTS to SCS, though the results of this comparison are yet to be published or presented.

### 4.1. GUARDIAN-Heart Registry

The GUARDIAN-Heart registry trial was launched in 2015 to assess the clinical impact of the SCTS compared to the standard of care SCS for donor heart preservation. Over 1900 patients have been enrolled across 26 transplant centers in the United States and Europe. The trial includes both adult and pediatric candidates undergoing primary isolated heart transplantation, with primary outcomes focused on 2-year post-transplant survival, PGD, and the need for post-transplant MCS [44].

#### 4.1.1. Primary Outcomes of the GUARDIAN-Heart Registry

The most recent analysis of 2-year post-transplant survival revealed that the SCTS demonstrated superiority over SCS (95% vs. 89%, *p* = 0.04) in a propensity score-matched comparison, which included 353 patients in each group. Silvestry et al. attributed this difference to a lower incidence of severe PGD (5.4% vs. 11.0%, *p* = 0.009) and reduced post-transplant MCS utilization (20.7% vs. 27.5%, *p* = 0.043) in the SCTS group [43]. This is a landmark result in demonstrating the survival benefit of the SCTS, as prior analyses of the GUARDIAN-Heart registry have not observed a survival difference (Table 2) [44,45,46].

Since the early reports of the GUARDIAN-Heart registry trial, the SCTS has consistently been associated with reduced rates of severe PGD compared to SCS [46]. Initial findings by Shudo et al. demonstrated a threefold difference in severe PGD rates between SCTS and SCS (4.0% vs. 12.0%, *p* = 0.011), which persisted in unmatched multivariable regression (OR 3.384, 95% CI [1.28–8.96], *p* = 0.014). These results were confirmed by two additional analyses, demonstrating lower rates of severe PGD with SCTS (5.7% vs. 16.1%, *p* = 0.012) and (6.0% vs. 12.1%, *p* = 0.018) [44,45]. D’Alessandro et al. further investigated how the severity of PGD impacts post-transplant survival, demonstrating a significant decline in 1-year post-transplant survival from no PGD to moderate PGD and severe PGD (95.6% vs. 92.5% vs. 68.8%, *p* < 0.001 pairwise comparisons) [44]. Therefore, they hypothesized that this difference would drive an overall survival difference between the preservation methods, which was later confirmed by Silvestry et al. [43].

Similarly to the definition of severe PGD, post-transplant MCS has also been used as an indicator of graft preservation. The use of new temporary ventricular assist devices (VADs), ECMO, and IABP following transplantation have all been studied under the GUARDIAN-Heart Trial. The previously described studies all examined DBD heart transplantation and demonstrated that the rates of new ECMO or temporary VAD post-transplant are at least two-fold higher for SCS (10.4–26.4%) compared to the SCTS (5.4–8.2%, all *p* < 0.05) [43,45,46,47,49] (Table 2). However, conflicting results regarding the rates of IABP usage among SCTS and SCS cohorts have been reported. Only Voigt et al. have reported a greater proportion of SCS-preserved hearts requiring new IABP after transplantation (13.2% vs. 6.0%, *p* = 0.006) in a propensity score-matched cohort [45]. However, the remaining studies observed similar rate of IABP usage between the SCTS and SCS groups (6.3–10.7% vs. 9.0–14.6%, all *p* > 0.05) [44,46,48,49]. The consistent reduction in the need for new temporary VADs and ECMO is a promising outcome for the SCTS. These lifesaving devices carry additional operational risks, particularly in cases requiring long-term support. Moreover, the economic impact of these life support systems on both patients and the healthcare system is an important consideration.

To assess the economic impact of each preservation method, Voigt et al. utilized Medicare cost reports to analyze the overall costs associated with post-transplant hospital length of stay. Their findings revealed that, despite the approximately USD 15,000 cost of the SCTS, the mean total cost of post-transplant care was significantly lower compared to SCS, with a reduction of nearly USD 30,000 (USD 45,613 ± USD 36,653 for SCTS) compared to (USD 71,307 ± USD 99,998, *p* = 0.03) [44]. This reduction is likely driven by the higher utilization of MCS and the significantly longer ICU length of stay in the SCS cohort [45,48]. These results highlight the economic and clinical advantages of the SCTS over SCS.

#### 4.1.2. High-Risk Donor and Recipient Profiles

Despite the superior outcomes of the SCTS compared to SCS in matched cohort analyses, further examination of what donor and recipient characteristics benefit most from this advanced preservation method is necessary to optimize SCTS utilization practice. The large amount of data collected by the GUARDIAN-Heart registry has allowed multiple studies to examine the impact of the SCTS with ECDs, prolonged ischemic times, and MCS BTT modalities on post-transplant outcomes.

First, Moayedifar et al. studied outcomes from ECD hearts preserved with either the SCTS or SCS. Among these donors, total ischemic greater than 4 h was the predominant condition for extended criteria in the SCTS cohort compared to SCS (SCTS 67.9% vs. SCS 46.7%, *p* < 0.001), while the rate of all other extended criteria parameters (donor age greater than 55 years, left ventricular ejection fraction 40–50%, luminal irregularities, etc.) was similar to SCS (all *p* > 0.05). Longer total ischemic time in the SCTS cohort is consistently reported in the GUARDIAN-Heart database, and despite this, the rates of moderate PGD, severe PGD, and all post-transplant MCS were lower in the unmatched SCTS cohort [47]. Multiple studies have shown that the SCTS attenuates cold ischemic injury, as logistic regression analysis by Lerman et al. and D’Alessandro et al. found that up 7 to h of preservation time with the SCTS is associated with a lower risk of PGD than 3 h of SCS [33,49]. Farhoud et al. performed a similar analysis where donor hearts preserved with SCS for less than 3 h were compared to donor hearts preserved with the SCTS for more than 4 h, the rate of severe PGD remained nominally higher for SCS compared to the SCTS (SCTS 5.9% vs. SCS 11.8%, *p* = 0.28), and no difference in 30-day survival was observed [50]. These results are promising for the application of the SherpaPak in the case of longer expected ischemic time, such as further donor hospital distances and complex recipient procedures, including durable LVADs or congenital heart defects.

From a recipient perspective, an important risk factor to evaluate is the use of MCS as a BTT. In the current era of heart transplantation in the United States, these devices are frequently utilized to support critically ill patients and provide them with the highest priority for an available donor heart. Silvestry et al. evaluated how various MCS bridging modalities were related to post-transplant outcomes when the SCTS or SCS methods were used to preserve the donor’s heart. While the typical metrics of 1-year survival rate and severe PGD were similar in the MCS BTT cohort, it was notable that the SCTS reduced the need for post-transplant MCS and the rate of right ventricular dysfunction [48,49]. A more focused analysis by Lerman et al. analyzed the durable LVAD population and observed that the SCTS reduces the risk of severe PGD by nearly 3-fold (AOR 0.31, [95%CI, 0.13–0.85], *p* = 0.009). Given that durable LVAD as a BTT has independently been associated with an increased risk of severe PGD, this result demonstrates the value of the SCTS for especially vulnerable patient populations.

#### 4.1.3. Limitations of the SCTS GUARDIAN-Heart Registry

Several limitations exist for all studies utilizing the GUARDIAN-Heart registry data. First, the registry database is subject to errors related to data entry quality and completeness. Second, the decision to employ either SCS or the SCTS is at the discretion of the transplant physicians; therefore, the impact of potential bias in the selection of SCS and SCTS patients cannot be eliminated. Third, the included patients are from multiple transplant centers and multiple countries. The impact of institutional and national protocols, standard practice, and different population characteristics have an unknown impact on the results of this trial. Fourth, the patients were enrolled in the GUARDIAN-Heart trial in a ‘reverse consecutive’ manner. This means that a significant proportion of the SCS control cases were performed up to 3 years earlier than the SCTS cases. This is highly relevant, as the 2018 heart allocation change in the United States significantly impacted transplant practices in the country. This was reported by Silvestry et al., where 74.9% of SCS recipients were transplanted after the 2018 policy change compared to 98.3% of the SCTS cohort (*p* < 0.001) [48]. Furthermore, temporal differences in center experience, transplant protocols, and the type of heart preservation solution used may impact post-transplant outcomes.

These studies leveraged multiple strategies to manage and minimize the potential bias associated with these limitations. Propensity score matching and multivariable regression were common statistical methods used to control for differences in the donor and recipient populations. This analysis consistently showed that the SCTS was used to preserve donor hearts with longer ischemic times and travel distances. Furthermore, the change in the United States’ allocation policy was reflected in shorter waitlist times for the SCTS cohort. Randomized control trials and additional GURADIAN-Heart registry studies with a larger sample and longer follow-up period are required to validate the positive effect of SCTS.

### 4.2. Single-Center Experience with the SCTS

In addition to the registry-based studies, single-center experiences are useful for studying the impact of the SCTS compared to SCS. Multiple centers around the world have contributed to this body of literature, and the opportunity to evaluate more granular hemodynamic, histological, and biochemical data allows for a more in-depth analysis of controlled hypothermic storage.

The first clinical use of the SCTS was published in 2020, and the early case series established the appropriate methods for future implementation of this device around the world [42,51,52]. Studies by Kawabori et al. and Schmiady et al. revealed an important limitation of the SCTS [53,54]. This device relies on phase-changing cooling packs to maintain temperature. No active cooling or refrigeration is present to decrease the weight, cost, and complexity of the disposable unit. However, if the initial temperature of the cardiac preservation solution is above the recommended temperature range, the SCTS cannot account for this difference. This will result in prolonged exposure to temperatures greater than 8 °C, predisposing the graft to ischemic injury. Next, large single-center retrospective reports first reported the lower rates of PGD with the SCTS, serving as a prelude to the GUARDIAN-heart trial results [55,56,60].

Examinations of granular single-center data with the SCTS were performed by Alam et al. and Lotan et al. First, Alam et al., analyzed the levels of cell-free donor-derived DNA (cf-ddDNA) in the recipient as a surrogate of graft health [57]. Compared to SCS, there was no improvement in cf-ddDNA levels when using the SCTS, and the release was attenuated by younger donor age and decreased ischemic time. Lotan et al. evaluated the grafts at their center for ischemia-reperfusion injury (IRI) by the histological examination of coagulative myocyte necrosis [58]. This parameter was examined at 1, 4, and 8 weeks post-transplant. No difference was observed between the SCTS and SCS, but the overall ischemic time was longer in the SCTS cohort. Future studies are required to examine the clinical impact of these diagnostic methods, but they provide valuable insight into the potential mechanisms by which the SCTS offers the superior preservation observed from the GUARDIAN-Heart data [58].

Finally, a single study applied the SherpaPak CTS to DCD heart transplantation. An analysis by Urban et al. utilized thoracoabdominal normothermic regional perfusion (TA-NRP) to rapidly resuscitate and evaluate donor hearts prior to procurement and cold storage with the SCTS, up to 3.5 h. This study shows that the initial results with prolonged hypothermic storage of DCD hearts procured with TA-NRP are feasible, and preservation times can be extended without necessitating the use of the OCS Heart normothermic perfusion platform [59].

### 4.3. Conclusion and Remaining Questions with the SCTS

The advantage that the SCS provides in better preserving donor hearts, especially for at-risk recipient populations, is clear from the reduced incidence of PGD and superior survival. This was achieved by simple temperature control without coronary perfusion, highlighting the fact that standard SCS results in storage temperatures lower than the commonly accepted 4 degrees Celsius. However, multiple questions remain regarding the optimal use case and future directions of the SCTS. (1) What is the safe limit of static temperature-controlled storage with the SCTS? (2) What is the impact of the preservation solution composition in the SCTS? (3) Would a reusable refrigeration system with a precise temperature control system offer the same benefits with decreased disposable costs and lower sensitivity to initial conditions?

## 5. XVIVO Heart Preservation System

**Figure 4:**
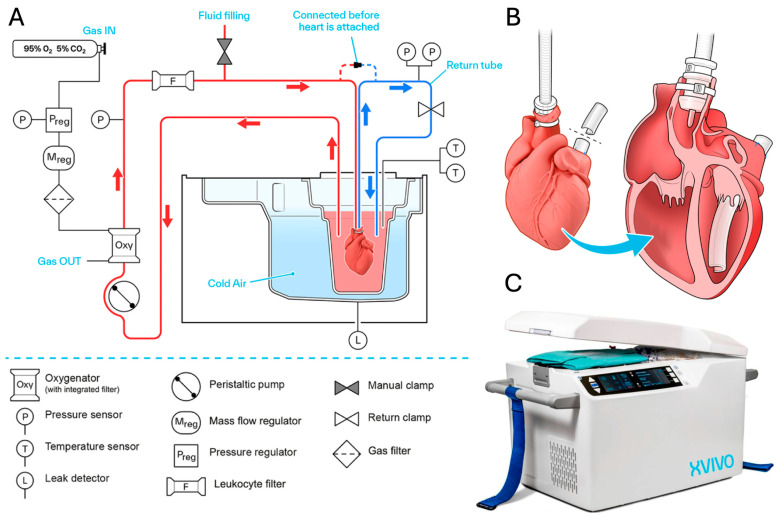
Diagram of the XVIVO Heart Preservation System with the (**A**) circuit diagram with perfusion and de-airing line as well as component labels, (**B**) heart preparation with novel de-airing cannula and left ventricular vent, and (**C**) image of the system exterior with control panel and information display [62].

The XVIVO Heart Preservation System from XVIVO Inc. (Gothenburg, Sweden) uses hypothermic oxygenated perfusion to preserve donor hearts suspended in a cardioplegic solution. The XHPS perfusion circuit includes a reservoir, a pressure-controlled roller pump, an oxygenator, and an arterial-leukocyte filter (Figure 4). The unit includes temperature control, sensor readings, and onboard oxygen and carbon dioxide sweep gas cylinders with a total weight of 32 kg. The XHPS reservoir contains 2.5 L of perfusion solution and ~500 mL of donor/recipient-compatible irradiated and leukocyte-reduced blood (from a hospital blood bank) to obtain a hematocrit of ~15% and a pH of 7.4. Antegrade perfusion through the aortic root is controlled to 20 mmHg, ranging between 150 and 250 mL/min [61].

### 5.1. Clinical Trials Evaluating the XHPS

The majority of clinical evidence studying the XHPS derives from clinical trials performed in Australia, New Zealand, and Europe. These studies have compared the outcomes of donor hearts preserved with the XHPS to SCS and focused on (1) non-inferior outcomes, (2) extending preservation times, and (3) a reduction in adverse cardiac events. In this section, we will discuss the results of the completed studies as well as the design of planned or ongoing clinical trials.

#### 5.1.1. NIHP Trial

The non-ischemic heart preservation (NIHP) trial, a non-randomized phase II study, was the first human use of the XHPS (Table 3) (NCT03150147). This study included 6 patients assigned to the XHPS experimental group and 25 assigned to the SCS control. The XHPS hearts were perfused for a median duration of 140 min [IQR 109–162 min]. All patients who received hearts preserved with the XHPS survived for 6 months after transplantation. No difference in early organ dysfunction was observed between the two groups, but nominally worse renal function was observed in the XHPS group based on the need for continuous renal replacement therapy (50% vs. 16%, RR 3.1, 95% CI [0.94, 10]). While this study did not utilize the completed version of the XHPS, it is the first demonstration of the safety and efficacy of prolonged hypothermic blood perfusion of donor hearts [61].

#### 5.1.2. HOPE Trial

Building upon the feasibility and safety of the XHPS established by the NIHP trial, a non-randomized, single-arm investigation using the XHPS with 36 heart transplant recipients across centers in Australia and New Zealand was performed by McGiffin et al. This study examined extended preservation times with the XHPS, between 6 and 8 h (Table 3) (ACTRN12620000595910). Recipient and donor inclusion criteria were open to institutional protocol, and the XHPS cohort was stratified by long preservation time (between 6 and 8 h) and short preservation time (<6 h). A comparator group of recipients in the ISHLT registry database undergoing DBD transplantation with SCS during the study period was used to benchmark the outcomes of these patients. Of the 36 donor hearts included, 29 were assigned to the long preservation group with a mean preservation time of 414 (±53) minutes, and 7 were included in the short preservation group with a preservation time of 252 (±55) minutes [62]. The primary conclusion of this study was that prolonged preservation, up to 9 h, was not associated with early graft failure of PGD. In the ISHLT DBD-SCS reference cohort, the 30-day mortality rate increased from 4% to 8% as graft ischemic time increased from 2 h to >5 h. However, no 30-day mortality was observed in the XHPS cohort, with a mean preservation time of 414 min (6.9 h) [62]. This indicates that the XHPS is capable of attenuating the adverse effects of prolonged cold ischemia

#### 5.1.3. NIHP 2019 Trial

A recent study by Rega et al. expanded on the work of McGiffin et al., comparing the outcomes between two groups of heart transplant recipients: 101 hearts preserved using HOPE with the XHPS, and 103 preserved via SCS across 15 transplant centers in Europe (Table 3) (NCT03991923) [66]. The primary outcomes included time to cardiac-related death, left or right PGD, acute cellular rejection, and graft failure requiring MCS or re-transplantation. The median preservation time of the XHPS group was slightly longer than that of the SCS cohort (240 [IQR 194–274] min vs. 215 [IQR 183–253] min), but no statistical comparison was reported. Although the overall hazard ratio indicates that XHPS did not significantly reduce the risk of all primary events compared to SCS (HR 0.56, 95% CI [0.32, 0.99]), the XHPS did result in a decreased risk of severe PGD (RR 0.24, 95% CI [0.10, 0.16]). No difference in the risk of 30-day cardiac death was observed between the groups (XHPS 2% vs. SCS 4%, HR 0.52, 95% CI [0.09–2.81]) [63]. To date, the results from these studies suggest that XHPS can effectively preserve donor hearts for extended durations. The study remains active but is no longer recruiting patients to continue studying outcomes up to one year after transplantation.

#### 5.1.4. Ongoing Clinical Trials

Multiple clinical trials utilizing the XHPS are currently underway worldwide. The First Clinical Evaluation of Heart Transplantation With Grafts Preserved Using an Ex vivo Extended Perfusion System (PEGASE) trial began in September 2023 at a single center in France to evaluate the safety and efficacy of the XHPS in preserving donor hearts for up to 14 h (NCT06035991, APHP220091) [67]. The primary outcome of this study is the recovery of cardiac function within 15 days after transplantation, defined as a cardiac index greater than 2.5 L/min/m^2^ without inotropic support or MCS. Secondary outcomes are 1-year post-transplant survival and freedom from graft-related adverse events [67].

The second trial is the Study of Hearts Transplanted After Non-Ischemic Heart PRESERVation from Extended Donors (PRESERVE) (NCT05881278). The PRESERVE trial is a prospective, multi-center, single-arm open-label study that began in October 2024 and includes 20 transplant centers in the United States [68]. Approximately 140 patients have been enrolled, and the estimated primary completion date is November 2025 with a 5-year follow-up complete in November 2029. This study evaluates ECDs, defined as (1) DBD donors with an expected cross-clamp time ≥ 4 h; (2) DBD donors with an expected cross-clamp time ≥ 2 h and age ≥ 50 years, ejection fraction of 40–50%, mild hypertrophy (1.2–1.6 cm) or coronary abnormalities without significant coronary artery disease; (3) DCD donors. The primary outcomes are 1-year post-transplant survival and freedom from graft failure, defined as cardiac death or re-transplantation, within 30 days [68].

The third trial currently underway is the Hypothermic Oxygenated Perfusion of Donor Hearts After Circulatory Death (HOPE@Heart) using the XVIVO Heart Assist Transport System [69]. This trial includes a single center in Belgium and began in August 2024. Similar to the PRESERVE trial, this study aims to examine the efficacy of the XHPS for DCD heart transplantation. However, this study only includes DCD heart donors, and the primary outcomes are limited to a 6-month follow-up of patient survival and graft failure. Additional secondary endpoints are also included, namely the incidence of PGD, acute rejection, and hospital length of stay [69].

#### 5.1.5. Limitations of the XHPS Clinical Trials

The clinical trials with the XHPS have demonstrated that (1) this technology is safe and effective with normal donor and recipient risk profiles and standard preservation times; (2) heart allografts can be preserved for up to 9 h and remain viable for preservation; and (3) this technology can decrease the risk of adverse graft-related events, such as PGD. However, there are significant limitations to the conclusions presented by the completed trials. First, only early post-transplant outcomes have been evaluated, up to 6 months, as these recent studies have not had the opportunity to perform extended follow-up. Second, each study was limited by low patient numbers and the potential impact of varying practices among transplant centers, influencing the results in an unknown fashion. Finally, the XHPS was only compared to standard-of-care SCS. The ongoing clinical trials can indirectly compare normothermic and hypothermic heart perfusion with single-arm investigations in ECD and DCD heart transplantation. Still, no clinical trial comparing these two strategies is currently underway.

### 5.2. Single Center Experiences with the XHPS

Single-center experiences by centers involved in the various XHPS clinical trials have examined specific population strata and compared the XHPS to other advanced preservation systems. A single-center experience presented by Joshi et al. explores the use of both the OCS normothermic machine perfusion system and the XHPS across two distinct populations [64]. The OCS is utilized for procuring and evaluating DCD heart allografts, while the XHPS is employed for the extended preservation of DBD hearts. The results of this study demonstrate excellent outcomes with these advanced techniques, highlighting the future of advanced cardiac preservation modalities. Each system serves specific needs, whether for functional evaluation, advanced therapies in ex situ systems, or managing prolonged ischemic times. As access to OCS, XHPS, and SCTS expands globally, further analyses like this will help assess the impact of these novel systems on increasing access to heart transplantation.

Similarly, Brouckaert et al. reported a case series utilizing the XHPS for preserving DCD donor hearts, following direct procurement in three patients. Unlike the OCS, which facilitates functional evaluation of the graft, the XHPS was employed in situations where a short agonal phase and minimal functional warm ischemia time were present. This approach was used after completing thorough pre-donation diagnostic testing, aligning with the institution’s ethical framework. The functional warm ischemia times were 15, 17, and 21 min, and the respective perfusion times with XHPS were 277, 281, and 179 min. All patients were successfully transplanted, no PGD was observed, and they survived to follow-up at 110, 90, and 55 days post-transplant [65].

### 5.3. Conclusion and Remaining Questions with the XHPS

The current clinical evidence with the XHPS shows that this technology is capable of doubling the safe preservation time of standard SCS. These outcomes indicate that the residual cellular metabolism under hypothermic conditions is sufficient to benefit from perfusion with an oxygenated solution but does not result in the rapid accumulation of waste products that may damage a heart allograft during prolonged normothermic perfusion. The ongoing clinical trials with this system follow a similar path to the OCS Heart: examining the utility of the system for ECDs (PRESERVE Trial) and DCD heart transplantation (HOPE@Heart Trial), with ongoing investigation into the maximum perfusion time (PEGASE Trial). However, many questions still remain regarding the clinical utilization of this new heart preservation system. Can the XHPS be operationalized for pediatric populations to enable greater organ sharing in this small population? What biomarkers and perfusion conditions are predictive of graft function before transplantation?

## 6. Conclusions and Future Directions

Heart transplantation remains the gold standard therapy for appropriately selected patients with end-stage heart failure, and the demand for available donor hearts is expected to grow alongside the rising prevalence of cardiovascular diseases in an aging population. Optimal outcomes in heart transplantation depend on careful donor and recipient selection, as well as effective organ procurement and preservation. As transplant communities worldwide push the boundaries of ischemic time restrictions and conventional donor selection criteria, innovative preservation strategies must evolve to address the limitations of traditional SCS. These advancements are critical for enabling the safe transportation and implantation of donor hearts over longer distances and within broader sharing networks.

In this review, we discussed the clinical impact of novel organ perfusion systems, including the OCS and XHPS, and temperature control systems, including the SCTS. Collectively, these preservation systems facilitate broader sharing of available donor hearts and enable expansion of the donor pool to offer comparable or potentially improved post-transplant survival. Therefore, the next challenge with these systems is to determine the optimal use case and allocation of these limited resources. The recent experience of a single transplant center in Australia highlights this future, where SCS, the OCS Heart, and the XHPS are available to the heart transplant team [64]. In this study, all OCS heart runs were allocated to DCD donors, extended preservation of DBD hearts was assigned to the XHPS, and standard criteria transplantations were performed with SCS. As access to these systems continues to expand, it is important to monitor how centers effectively allocate these systems, as both normothermic and hypothermic preservation techniques offer distinct advantages.

As donor organ preservation and assessment techniques advance, the current definition of standard-criteria donors may prove overly restrictive. The clinical evidence with each preservation system shows that heart perfusion and temperature-controlled storage ameliorate risk factors for graft dysfunction. Many of these donor characteristics, including ventricular, coronary, or functional imperfections, would have resulted in the refusal of the organ for transplantation 10 years ago. Expanding these criteria could significantly enhance donor organ utilization, shorten waitlist times, and improve overall patient outcomes. Furthermore, ex vivo machine perfusion platforms may provide opportunities for donor organ-targeted therapies before transplantation. These interventions could mitigate ischemia–reperfusion injury and even improve the function of marginal hearts, making them suitable for transplantation. While these therapies are not yet clinically available, emerging evidence suggests considerable promise.

Another important consideration is the economic impact and feasibility of these advanced heart preservation systems. The cost and complexity of SCS is low, so the burden of proof lies on the advanced systems to demonstrate improved outcomes or extended applications. The results of the studies evaluating the impact of SCTS support this conclusion, demonstrating that the added post-transplant care burden from the higher complication rates associated with SCS, compared to SCTS, justifies the cost of the disposable temperature control system [45]. A similar analysis of two cases of extended heart preservation showed that the OCS results in lower overall costs compared to SCS, again due to a decrease in post-transplant adverse events [70]. These early results suggest that these systems offer true clinical and financial benefits to patients, but large-scale analysis is required, especially among varying national healthcare systems.

As organ perfusion and temperature control technologies continue to improve, the safe extension of heart ischemic times beyond the limitations of current SCS becomes increasingly feasible. This progress opens the possibility of delaying nighttime transplants to daylight hours. The prospect of elective heart transplantation holds the potential to reduce system-level fatigue among transplant teams, including surgeons, anesthesiologists, nurses, perfusionists, and intensive care staff, ultimately improving patient outcomes [71]. As preservation strategies evolve, the ability to reimagine the logistics and timing of heart transplantation may become a transformative aspect of the field.

## Figures and Tables

**Table 1:** Summary of the clinical trials and single center experiences grouped by topic, analyzing outcomes with the OCS Heart.

TransMedics Organ Care System Heart				
Clinical Trials				
*Study Information*			*Outcomes*	
Author, Year	Study Design	Patient Population	Primary Outcome	Secondary Outcome
Ardehali, et al. 2015 PROCEED-II Trial [9]	Prospective, open-label, multicenter randomized clinical trial	OCS (n = 63) versus SCS (n = 67) with standard criteria donors	Non-inferior 30-day survival (OCS 94% vs. SCS 97%, *p* = 0.36).	No difference in cardiac adverse events, severe rejection, or ICU length of stay (all *p* > 0.05).
Schroder, et al. 2024 EXPAND-CAP Trial [11]	Prospective, single-arm, multicenter clinical trial	OCS (n = 173) with ECDs	30-day survival free of severe PGD achieve in 92% of grafts ( > 65%).	Donor heart utilization rate (87%), severe graft-related events (17%), and severe PGD (16%).
Schroder, et al. 2023 DCD Heart [12]	Prospective, open-label, multicenter, randomized clinical trial	DCD-OCS (n = 90) versus DBD-SCS (n = 90)	6-month survival (DCD-OCS 94% vs. DBD-SCS 90%, *p* < 0.001 for non-inferiority).	Moderate or severe PGD (DCD-OCS 22% vs. DBD-SCS 10%).
**Single-Center Experiences: High Risk Donor & Recipient Populations**				
***Study Information***			***Outcomes***	
**Author, Year**	**Study Design**	**Patient Population**	**Primary Outcome**	**Secondary Outcome**
Garcia Saez et al., 2014, United Kingdom [13]	Descriptive case series	OCS (n = 26) with ECDs and/or high-risk recipients (MCS or elevated PVR)	100% 1-month survival and 96% survival at 257 ± 116 days of follow-up	Post-transplant IABP (11%), moderate right ventricular failure (19.2%).
Sponga et al., 2020, Italy [14]	Retrospective cohort	OCS (n = 21) versus SCS (n = 79) with ECDs (EXPAND trial criteria)	Post-transplant 30-day survival comparable between (OCS 100% vs. SCS 95%, *p* = 0.58), and estimated 5-year survival higher for OCS (73% vs. 100%, *p*: 0.04), with incomplete follow-up (median follow-up CSS: 32 and OCS: 19).	Lower incidence of early post-transplant complications (PGD, need for ECMO, atrial fibrillation, renal or respiratory insufficiency, acute rejection) for OCS (67% vs 87%, *p*: 0.04)
Sponga et al., 2023, Italy [15]	Case series	OCS (n = 80) with ECDs and high-risk recipients	Post-transplant 1-year survival: 83 ± 4%, 3-year survival: 72 ± 7% (median follow-up: 16 months).	Rate of in-hospital mortality 9 (11%), moderate to severe PGD: 13 (16%).
Rojas et al., 2022, Germany [16]	Retrospective cohort	OCS (n = 68) versus SXS (n = 51 with ECDs and/or high-risk recipients	Difference in 30-day (OCS: 92.4%, CSS: 90.2%) and 1-year (OCS:89%, CSS: 85%) post-transplant survival not statistically significant between OCS and CSS.	Lower incidence of post-transplant dialysis in the OCS group (4.4% vs 27.5%). Comparable incidence of PGD requiring ECMO between OCS and CSS.
Sponga et al., 2021, Italy [17]	Retrospective cohort	OCS (n = 14) versus SCS (n = 24) with recipients MCS	Post-transplant 30-day mortality not significantly different between CSS (13%) and OCS (0%), *p* = 0.28.	PGD higher in CSS (42%) vs OCS (7%), *p* = 0.03.
Kaliyev et al., Kazakhstan, 2020 [18]	Retrospective cohort	OCS (n = 25) versus SCS (n = 10) with recipients on MCS	Post-transplant 30-day mortality not significantly different between CSS (100%) and OCS (96%), *p* = 0.5.	
Stamp et al., 2015, Australia [19]	Case report	Heart transplant after 10-hour preservation with OCS.	Discharged from hospital alive	Need for ECMO post-transplant
Medressova et al., 2021, Kazakhstan [20]	Case report	Heart transplant after 17-hour preservation with OCS.	Discharged from hospital alive	Need for ECMO post-transplant
Dang Van et al., 2021, France [21]	Descriptive case series	DBD-OCS (n = 4) with ECDs and DCD-OCS (n = 1) OCS	Post-transplant 3-month survival: 75%.	Post transplant ECMO: 3 (60%).
Fleck et al., 2021, Germany [22]	Retrospective cohort	OCS (n = 8) versus SCS (n = 13) for pediatric heart transplantation	Post-transplant 1-year survival similar between CSS (85%) and OCS (88%).	Incidence of PGD, renal failure, and hepatic failure similar between OCS and CSS.
Verzelloni et al., 2021, United Kingdom [23]	Descriptive case series	OCS (n = 9) for recipients with adult congenital heart disease undergoing heart transplantation	Post-transplant 30-day survival: 89%, 1-year survival: 78%.	Post-transplant ECMO in 44%.
Isath et al., 2023, United States [24]	Retrospective cohort	OCS (n = 8) versus SCS (n = 13) with expected transport time >4 h.	Post-transplant in-hospital survival similar between OCS (100%) and CSS (92%), *p* = 0.85.	PGD similar between OCS (12%) and CSS (15%), *p* = 0.85.
Pizanis et al., 2023, Germany [25]	Descriptive case series	OCS (n = 12) with ECDs	Post-transplant 30-day survival: 100%.	Post-transplant graft LVEF >50% in all cases, no cases of rejection on endomyocardial biopsies.
Medina, et al. 2024, United States [26]	Descriptive case series	OCS (n = 8) for pediatric heart transplantation	100% survival (median follow-up 11.9 months)	50% (n = 4) required either post-operative VAD or ECMO support.
**Single-Center Experiences: DCD Heart Transplantation**				
***Study Information***			***Outcomes***	
**Author, Year**	**Study Design**	**Patient Population**	**Primary Outcome**	**Secondary Outcome**
Dhital et al., 2015, Australia [27]	Case series	OCS (n = 3) for DCD heart transplantation	All patients survived past 2-months postop.	Post-transplant mechanical circulatory support: 2 (66%).
Mehta et al., 2019, United Kingdom [28]	Case series	OCS (n = 7) for DCD heart transplantation	Post-transplant 30-day survival: 100%, 90-day survival: 86%.	Post-transplant ECMO: 3 (43%) Initiation of the OCS DCD program resulted in 23% increase in heart transplant volume.
Cernic et al., 2022, United Kingdom [29]	Case-control	OCS (n = 51) for DCD heart transplantation stratified by need for post-transplant MCS (MCS n = 20 versus No MCS n = 31)	OCS lactate levels, arteriovenous lactate gradient, or the percentage of patients with lactate >5 mmol/L at 3 h were significantly associated with the need for post-transplant MCS.	
Coniglio et al., 2023, United States [30]	Retrospective cohort	DCD-OCS (n = 31) versus DBD-OCS (n = 16) versus DBD-SCS (n = 38)	No difference in post-transplant 6-month survival between the transplant types.	Higher incidence of PGD requiring MCS in the DCD group. No difference in cardiac MRI findings between the transplant types.
**Single-Center Experiences: Advanced Uses**				
***Study Information***			***Outcomes***	
**Author, Year**	**Study Design**	**Patient Population**	**Primary Outcome**	**Secondary Outcome**
Ghodsizad et al., 2012, Germany [31]	Case report	DBD heart evaluated with coronary angiography during OCS perfusion	Successful diagnostic ex-vivo coronary angiography.	
Meredith et al., 2022, Australia [32]	Case series	DCD-OCS (n = 8) undergoing coronary angiography during OCS support	Successful ex-vivo coronary angiography in all cases without injury to the graft.	Six hearts (75%) proceeded to transplantation. Severe multivessel disease found in one graft (12.5%), all other hearts were found to have <30% coronary stenosis.
Krishnan et al., 2024, United States [33]	Retrospective cohort	OCD Beating Heart DCD (n = 21) and DBD (n = 3) versus OCD-DCD (n = 22)	Post-transplant survival 95.8% at median 192-day follow-up in the beating heart transplant group.	Lower rate of post-transplant mechanical circulatory support in beating heart transplant vs. non-beating heart (0% vs. 36.4%, *p* < 0.005).

Abbreviations: Organ Care System (OCS), Static cold storage (SCS), Intensive care unit (ICU), Donation after circulatory death (DCD), Donation after brain death (DBD), Extended criteria donors (ECDs). Primary graft dysfunction (PGD), Mechanical circulatory support (MCS), Pulmonary vascular resistance (PVR).

**Table 2:** Summary of the GUARDIAN-Heart registry trials and single center experiences grouped by topic analyzing outcomes with the SherpaPak Cardiac Transport System.

Paragonix SherpaPak Cardiac Transport System				
GURADIAN-Heart Registry: Primary Outcomes				
*Study Information*			*Outcomes*	
Author, Year	Study Design *	Patient Population	Post-transplant Survival	Severe PGD
Shudo, et al., 2023 [46]	Unmatched	SCS (n = 314) versus SCTS (n = 255)	SCS (90.6%) versus SCTS (92.6%, p = 0.37) after 1 year.	SCS (10.2%) versus SCTS (5.4%, *p* = 0.03).
	Propensity score-matched	SCS (n = 150) versus SCTS (n = 150)	SCS (88.7%) versus SCTS (94.0%, p = 0.10) after 1 year.	SCS (12.0%) versus SCTS (4.0%, *p* = 0.011).
Voigt, et al., 2023 [24]	Propensity score-matched	SCS (n = 87) versus SCTS (n = 87)	SCS (86.2%) versus SCTS (92.0%, *p* = 0.23) after 1 year.	SCS (16.1%) versus SCTS (5.7%, *p* = 0.03).
D’Alessandro, et al., 2024 [44]	Propensity score-matched	SCS (n = 281) versus SCTS (n = 281)	SCS (92.1%) versus SCTS (95.9%, *p* = 0.07) after 1 year.	SCS (12.1%) versus SCTS (6.0%, *p* = 0.018).
Silvestry, et al., 2024 [43]	Propensity score-matched	SCS (n = 353) versus SCTS (n = 353)	SCS (89.9%) versus SCTS (94.9%, *p* = 0.021) after 2 years.	SCS (11.0%) versus SCTS (5.4%, *p* = 0.009).
**GUARDIAN-Heart Registry: Donor and Recipient Risk Stratification**				
***Study Information***			***Outcomes***	
**Author, Year**	Study Design *	**Patient Population**	**Post-transplant Survival**	**Severe PGD**
Moayedifar, et al., 2024 [47]	Extended criteria donors, unmatched	SCS (n = 137) versus SCTS (n = 193)	SCS (89.6%) versus SCTS (92.9%, *p* = 0.41) after 1 year.	SCS (13.9%) versus SCTS (6.2%, *p* = 0.022).
Silvestry, et al., 2024 [48]	MCS BTT, unmatched	SCS (n = 354) versus SCTS (n = 422)	SCS (91.5%) versus SCTS (93.5%, *p* = 0.18) after 1 year.	SCS (10.2%) versus SCTS (6.2%, *p* = 0.046).
	MCS BTT, PSM	SCS (n = 216) versus SCTS (n = 216)	SCS (92.5%) versus SCTS (93.2%, *p* = 0.757) after 1 year.	SCS (10.2%) versus SCTS (5.1%, *p* = 0.069).
Lerman, et al., 2024 [49]	LVAD BTT, unmatched	SCS (n = 178) versus SCTS (n = 149)	SCS (94.4%) versus SCTS (96.6%, *p* = 0.492) after 1 year.	SCS (14.0%) versus SCTS (5.4%, *p* = 0.010)
	LVAD BTT, PSM	SCS (n = 216) versus SCTS (n = 216)	SCS (93.2%) versus SCTS (90.2%, *p* = 0.57) after 1 year.	SCS (15.5%) versus SCTS (3.6%, *p* = 0.016).
Farhoud, et al., 2024 [50]	SCS < 3 hours versus SCTS > 4 hours, unmatched	SCS (n = 183) versus SCTS (n = 148)	SCS (100.0%) versus SCTS (98.6%, *p* = 0.20) after 30 days	SCS (10.4%) versus SCTS (8.1%, *p* = 0.57)
	SCS < 3 hours versus SCTS > 4 hours, PSM	SCS (n = 85) versus SCTS (n = 85)	SCS (100.0%) versus SCTS (98.8%, *p* > 0.99) after 30 days	SCS (11.8%) versus SCTS (5.9%, *p* = 0.28)
**Single-Center Experiences**				
***Study Information***			***Outcomes***	
**Author, Year**	**Study Design**	**Patient Population**	**Primary Outcomes**	**Secondary Outcomes**
Naito, et al., 2020 [42]	Case report	SCTS (n = 1)	Discharged to home after 17 days with left ventricular ejection fraction of 72%.	
Radakovic, et. al., 2020 [51]	Case control, retrospective analysis	SCTS (n = 7) versus SCS (n = 14)	1 year survival (SCTS 71.4% vs. SCS 78.6%, *p* = 0.717).	Improved right heart function after 30-dau follow up (tricuspid annular plane systolic excursion DBD-SCTS 17.83 ± 2.71 vs. DBD-SCS 14.52 ± 2.61 mm, *p* = 0.02).
Guenthart, et al., 2021 [52]	Case report	SCTS (n = 1)	Discharged to home after 10 days, and 3-month complication-free survival	
Schmiady, et al., 2021 [53]	Single-arm, retrospective analysis	SCTS (n = 4)	All patients survived to the end of follow up (average 303 days). One replicate was notable for warm preservation (>10 C) during preservation due to high initial temperature.	
Kawabori, et al., 2022 [54]	Case series	SCTS (n = 15)	SCTS (n = 3) notable for warm preservation (>10 C) due to high initial preservation solution temperature.	
Bitargil, et al., 2022 [55]	Retrospective analysis	SCTS (n = 34) versus SCS (n = 47)	No difference in 30-day survival (SCTS 100% vs. SCS 98%, *p* = 0.7).	No significant difference in groups regarding VIS, PGD, or the need for a temporary pacer.
Lechiancole et. al., 2024 [56]	Retrospective Analysis	SCTS (n = 30) versus SCS (n = 60)	1-year survival (SCTS 90% vs. SCS (88%, *p* = 0.89).	Rate of moderate to severe was similar (SCTS 7% vs. SCS 20%, *p* = 0.08).
Alam et. al., 2023 [57]	Retrospective analysis	SCTS (n = 35) versus SCS (n = 30)	Higher risk of elevated dd-cfDNA with SCS compared to SCTS (AOR 4.9, o5% CI [1.08, 22.5]. *p* = 0.046).	
Lotan et al., 2024 [58]	Single-blinded, retrospective analysis	STCS (n = 57) versus SCS (n = 33)	No significant differences in ischemic reperfusion injuries were observed between groups at weeks 1, 4, and 8 post-transplant.	A 59.3% reduction in coagulative myocyte necrosis occurred from weeks 1 to 4 with SCTS but not with SCS. Similar survival and rejection rate after 1 year.
Urban et al., 2023 [59]	Single-arm, retrospective analysis	DCD-SCTS (n = 12)	83% survival to discharge (n = 10) with one death from graft dysfunction (8%).	Median length of ICU stay for hospital survivors was 5 days and hospital stay 17 days.

* All studies utilizing the GUARDIAN-Heart registry database are retrospective observational cohort analyses. Abbreviations: Global Utilization And Registry Database for Improved heArt preservatioN (GUARDIAN), SherpaPak Cardiac Transport System (SCTS), Static cold storage (SCS), Donation after brain death (DBD), Vasoactive inotropic score (VIS), Primary graft dysfunction (PGD), Donor-derived cell-free deoxyribonucleic acid (dd-cfDNA), Adjusted odds ratio (AOR), intensive care unit (ICU).

**Table 3:** Summary of clinical trials and singe center experiences with the XVIVO Heart Preservation system.

XVIVO Heart Preservation System				
Clinical Trials				
*Study Information*			*Outcomes*	
Author, Year	Study Design	Patient Population	Primary	Secondary
Nilsson, et al., 2020 NIHP Trial [61]	Prospective, open-label, multicenter, non-randomized clinical trial	XHPS (n = 6) versus SCS (n = 353) with standard criteria donors	6-month survival without severe PGD, ECMO, or acute cellular rejection (XHPS 100% vs. SCS 72%, RR 1.4 95% CI [1.1–1.8]).	No significant differences: IR-injury, Immediate graft function, and 6-month adverse events.
McGiffin, et al., 2024 HOPE Trial [62]	Prospective, single-arm, multicenter, non-randomized clinical trial	XHPS (n = 7) short preservation (<6 h) versus XHPS (n = 29) long preservation (6–9 h)	100% survival in both cohorts at 30 days.	One instance of PGD in the long preservation cohort.
Rega, et al., 2024 NIHP2019 [63]	Prospective, open-label, multicenter, randomized, clinical trial	XHPS (n = 101) versus SCS (n = 103)	Composite cardiac-related death, PGD, rejection, or graft failure (XHPS 19% vs. SCS 30%, *p* = 0.059).	No difference observed in the rate of serious adverse events.
**Single Center Experiences**				
***Study Information***			***Outcomes***	
**Author, Year**	**Study Design**	**Patient Population**	**Primary**	**Secondary**
Joshi, et al., 2024 [64]	Retrospective analysis	DCD-NMP (n = 44) versus BD-HMP (n = 38) versus BD-SCS (n = 78)	No difference in severe PGD or 30-day survival (DCD-NMP 100% vs. BD-HMP 97% vs. BD-SCS 100%).	
Brouckaert et al., 2024 [65]	Prospective, single arm case series	DCD-XHPS donors (n = 3)	No severe PGD in any recipients and 100% survival at 30 days.	

Abbreviations: XVIVO Heart Preservation System (XHPS), Static cold storage (SCS), Primary graft dysfunction (PGD), Extracorporeal membrane oxygenation (ECMO), Ischemia-reperfusion (IR).

## Data Availability

No new data were created or analyzed in this study. Data sharing is not applicable to this article.

## Abbreviations

AOR	Adjusted odds ratio
BTT	Bridge to transplant
CAV	Cardiac allograft vasculopathy
CFDD-DNA	Cell-free donor-derived deoxyribonucleic acid
CE	European Conformity
CI	Confidence interval
DBD	Donation after brain death
DCD	Donation after circulatory death
ECD	Extended criteria donors
ECMO	Extracorporeal membrane oxygenation
EXPAND	Expanded Criteria Donor Hearts for Transplantation
EXPAND-CAP	Expanded Criteria Donor Hearts for Transplantation Continued Access Protocol
FDA	Food and Drug Administration
GUARDIAN	Global Utilization And Registry Database for Improved heArt preservation
HOPE	Hypothermic oxygenation machine perfusion
IABP	Intra-aortic balloon pump
ICU	Intensive care unit
IQR	Interquartile range
IVUS	Intravascular ultrasound
ISHLT	International Society of Heart and Lung Transplantation
LVAD	Left ventricular assist device
MCS	Mechanical circulatory support
MRI	Magnetic resonance imaging
NF-MACE	non-fatal major adverse cardiovascular event
NIHP	Nonischemic heart preservation
OCS	Organ care system
OR	Odds ratio
PGD	Primary graft dysfunction
PROCEED-II	Ex vivo Perfusion of Donor Hearts for Human Heart Transplantation
RR	Risk ratio
SCS	Static cold storage
SCTS	SherpaPak cardiac transport system
TA-NRP	Thoracoabdominal normothermic regional perfusion
UNOS	United Network for Organ Sharing
VAD	Ventricular assist device
XHPS	XVIVO Heart Preservation System
